# Molecular Characterization of *CnHd3a* and Spatial Expression of Its Alternative Splicing Forms Associated with Flowering Transition and Flower Development in Coconut Palm (*Cocos nucifera* L.)

**DOI:** 10.3390/genes16060718

**Published:** 2025-06-18

**Authors:** Pariya Maneeprasert, Siriwan Thaisakun, Theerachai Thanananta, Narumol Thanananta, Noppamart Lokkamlue, Chareerat Mongkolsiriwatana

**Affiliations:** 1 Interdisciplinary Graduate Program in Genetic Engineering and Bioinformatics, The Graduate School, Kasetsart University, 50 Ngamwongwan, Chatuchak, Bangkok 10900, Thailand; pariya.man@ku.th (P.M.); siriwan.tha@ncr.nstda.or.th (S.T.); thana@tu.ac.th (T.T.); narumol@vru.ac.th (N.T.); 2 National Center for Genetic Engineering and Biotechnology, National Science and Technology Develoment Agency, Khlong Nueng, Khlong Luang, Pathum Thani 12120, Thailand; 3 Department of Biotechnology, Faculty of Science and Technology, Thammasat University, Rangsit Centre, Khlong Nueng, Khlong Luang, Pathum Thani 12120, Thailand; 4 Faculty of Science and Technology, Valaya Alongkorn Rajabhat University under Royal Patronage, Khlong Nueng, Khlong Luang, Pathum Thani 13180, Thailand; 5 Department of Science and Bioinnovation, Faculty of Liberal Arts and Science, Kasetsart University, Kamphaeng Saen Campus, Nakhon Pathom 73140, Thailand; faasnmlo@ku.ac.th

**Keywords:** alternative splicing, *CnHd3a*, coconut palm, molecular characterization, flowering transition

## Abstract

Background: The flowering transition is a critical process determining the onset of reproductive development and fruit production. The molecular mechanisms underlying this process in coconuts are poorly understood; however, recent studies have identified *CnHd3a* as a potential regulator of the floral transition in coconuts. Methods: In this study, we characterized the molecular structure of *CnHd3a* and analyzed its alternative splicing forms in tall and dwarf varieties of coconut palms during the flowering transition. We used qRT-PCR to measure the expression levels of *CnHd3a* at different developmental stages. Results: *CnHd3a* was expressed in leaves and the shoot apical meristem (SAM) during the flowering transition in both coconut varieties and flower tissues during flower development. Interestingly, the expression levels of complex isoforms of *CnHd3a* were higher in the leaves of dwarf coconuts than in those of tall coconuts, suggesting their involvement in shortening the vegetative growth phase of dwarf coconuts. The gene structure of *CnHd3a* was found to be conserved across different plant species, indicating the evolutionary conservation of the floral transition process. Conclusions: Our findings provide insight into the molecular mechanisms underlying the floral transition and flower development processes in coconut palm. The tissue-specific expression patterns of *CnHd3a* isoforms show their potential roles in growth and development. Further investigations focusing on the functional characterization of *CnHd3a* isoforms will have practical implications for coconut breeding and cultivation strategies.

## 1. Introduction

The coconut palm (*Cocos nucifera*, 2n = 32) is a member of the Arecaceae family and the only species in the *Cocos* genus. It is cultivated in over 93 countries across Central and South America, East and West Africa, Southeast Asia, and the Pacific Islands, covering over 12 million hectares [1,2]. Given the economic importance of coconut production, it is crucial to understand the complex physiological process of the flowering transition in coconut palms [3,4]. The potential health benefits of coconut oil, including its ketogenic, glycemic, and atherogenic properties, further highlight the need for additional investigations into flowering transition and its regulation and the impact of these factors on breeding, coconut yield, and quality [5,6,7].

The flowering transition, which involves the differentiation of a shoot into an inflorescence, of coconut palms is a complex physiological process that marks a plant’s shift from vegetative to reproductive growth and is influenced by various factors, such as temperature, photoperiod, and hormonal regulation [8]. Coconut palm is classified into tall and dwarf types based on morphological characteristics and breeding habits [9]; tall coconut palms exhibit cross-fertilization (flowering transition around 8–10 years after planting), while dwarf coconut palms exhibit self-fertilization (flowering transition at 4–6 years after planting). The lengthy vegetative growth phase of this Palmae species before transitioning to flowering and fruiting constrains the conventional breeding progress and limits coconut production economics.

The flowering transition is primarily regulated by the action of *FLOWERING LOCUS T (FT)-like* genes, which integrate external and internal signals to control the process [10,11,12,13]. *FT* is a member of the *FT/TFL1* gene family, which contains the phosphatidyl ethanolamine-binding protein (PEBP) domain found in all taxa, including bacteria, mammals, and plants [14,15,16]. Several members of this gene family regulate the flowering transition and other plant developmental processes [17]. FT serves as a long-distance floral transition signal and is transported from the leaves to the shoot apical meristem (SAM) where flowering occurs [18,19,20]. *FT* is primarily expressed in leaves during flowering induction, and FT proteins move from leaves to the SAM to initiate flowering [21]. At the SAM, the FT protein forms a complex with the basic leucine zipper (bZIP) protein Flowering locus D (FD) and 14-3-3 protein, leading to the upregulation of floral meristem identity genes like *Apetala1 (AP1)* [22,23]. Therefore, because there is a strong correlation between *FT* expression levels and flowering onset [24], FT is used as a universal floral transition indicator in angiosperms.

Much of the understanding of the molecular control of flowering comes from studying model plants, such as *Arabidopsis (Arabidopsis thaliana)* and rice (*Oryza sativa L.*), annual plants that flower in response to long-day and short-day photoperiods, respectively [25]. The function of *FT* has been widely studied in various plant species, with diverse results. For instance, 3 out of 13 *FT-like* genes were found to promote flowering in rice, with *OsFTL2* identified as a mobile signal inducing flowering [26]. In barley, five *FT-like* genes showed differential expression patterns under varying conditions, indicating a potential regulatory complexity [27]. In contrast, only one of the four *FT-like* genes were shown to promote seasonal flowering in poplar [28,29].

Perennial tree species differ from annual plants in that they require several years of vegetative growth before transitioning to the reproductive stage, which is marked by their ability to form flower buds. In perennial woody plants, it has been reported that *FT* genes play key roles in manipulating flowering time, shortening the juvenile phase, and serving as valuable research and breeding tools [30]. *FT* homolog genes have been studied in various perennial plant species with long juvenile phases, such as Populus [25,29,31,32,33], apple (*Malus domestica*) [34], and citrus [35,36,37]. However, it should be noted that *FT* homologs from different plant species respond differently to external environmental factors and have varied functions in regulating flowering [38]. The *FT-like MdFT1* and *MdFT2* genes of apple (*M. domestica*) have also exhibited the potential to act as floral promoters [39,40]. The *FT* homolog (*CsFT*) of citrus regulates floral induction and promotes flowering in trifoliate orange (*Poncirus trifoliata*) [37].

As stated above, the flowering transition of coconut palms is regulated by various factors such as temperature, photoperiod, and hormonal regulation. In short and tall plants, the flowering transition may vary due to genetic and environmental factors. Short plants may transition to flowering faster than tall plants because of differences in light and hormone distributions. Additionally, taller plants may require more resources to transition to flowering because of the increased distance between the leaves and shoot meristem. Therefore, understanding the factors that regulate the flowering transition of coconut palms and other plants can help optimize their growth and economic production. Several key genes in the photoperiod pathway were differentially expressed between seedling and reproductive leaf samples in both tall and dwarf coconuts, including *suppressor of overexpression of constans 1 (SOC1)*, *flowering locus T (FT)*, and *Apetala 1 (AP1)*. Little is known about the post-transcriptional regulation of *FT* homolog genes responsible for the flowering transition in coconut palms. This lack of knowledge is particularly concerning, given the importance of the flowering time trait in this long-lived agricultural tree species. In this regard, Xia et al. (2020) [41] recently conducted a seminal study and validated the alternative splicing of *CnFT* transcripts as the likely causal mechanism for the observed differences in flowering time between tall and dwarf coconut types [41].

This study aimed to investigate the molecular structure, gene organization, and 3D protein structure of *FT* homolog genes in tall and dwarf coconut palm varieties and to analyze these genes’ expression patterns. We also focused on the post-transcriptional regulation and alternative splicing analysis of the genes involved in controlling the flowering transition. The results of this study are expected to contribute to a better understanding of the complex process of flowering in coconut palms and provide valuable insights for developing breeding tools for coconut palms and other perennial plant species.

## 2. Materials and Methods

### 2.1. Plant Materials

**Figure 1:**
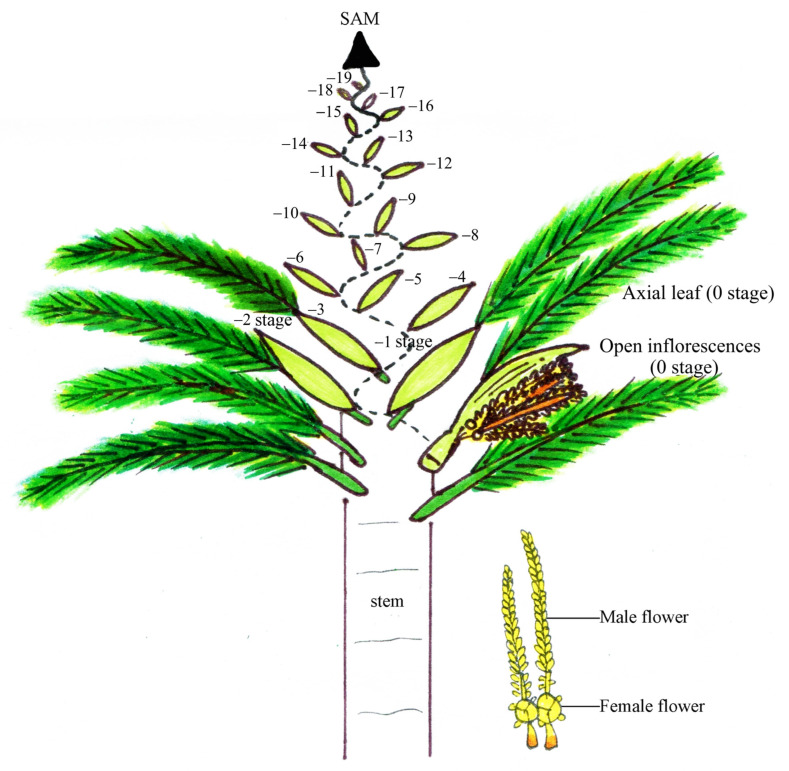
Various parts of coconuts collected for RNA extraction.

Dwarf and tall coconut palms were cultivated in a private experimental field in the Samut Sakhon Province, Thailand. Leaf and somatic apical meristem (SAM) samples were collected from various vegetative growth stages, including germination and 4, 6, 12 (1 year), and 24 months (2 years) after planting, as well as the reproductive growth stage displaying the first inflorescence at 36 months (3 years) for dwarf and 60 months (5 years) for tall palms. These samples were immediately immersed in liquid nitrogen and stored at −80 °C until needed. During the reproductive stage of the dwarf coconut palms, the first (0), second (−1), third (−2), and fourth (−3) inflorescences were collected, with male and female flowers from each stage separated. Axial leaves from each inflorescence stage, L (0), L (−1), L (−2), and L (−3), were also collected. All collected samples were immediately dipped in liquid nitrogen and stored at −80 °C for RNA extraction. The various tissue samples collected are shown in Figure 1.

### 2.2. DNA Extraction

Total genomic DNA was extracted from the young leaves of dwarf and tall coconut palms using the method described by Prasad et al. (2022) [42] and stored at −20 °C until needed. The quality and concentration of the DNA samples were assessed by 1% agarose gel electrophoresis and spectrophotometric analysis.

### 2.3. RNA Extraction and Reverse Transcription

Total RNA was extracted from three samples of each stage of coconut leaves and the SAM using the CTAB method [43] and lithium chloride precipitation as described by Stiekema et al. (1988) [44]. DNA was removed from the total RNA solution using DNase I (New England Biolabs Inc., Ipswich, MA, USA ). Five micrograms of DNaseI-treated RNA was converted to cDNA using SuperScript™ III Reverse Transcriptase (Invitrogen, Carlsbad, CA, USA) according to the manufacturer’s instructions. The reactions consisted of 1x reaction buffer, 5 µM oligo (dT) 20 primer, 5 mM DTT, 2 U RNaseOUT™ Recombinant RNase Inhibitor, 0.5 mM dNTP Mix, and 200 U SuperScript™ III-RT. The resulting cDNAs were used as the template for PCR amplification.

### 2.4. The Cloning of the Full-Length Genomic DNA and cDNA of CnHd3a

To isolate the *FT* homolog gene from dwarf and tall coconuts, degenerate primers designed by Hou and Yang (2009) [45] were used to amplify the PEBP conserved region using the touchdown PCR technique. Fifty nanograms of genomic DNA from dwarf or tall coconut was added to 20 µL of 1X PCR buffer containing 2.0 mM MgCl_2_, 0.2 mM dNTP, 0.5 µM each of CD.F and CD.R primers (Table 1), and 2U of Platinum™ Taq DNA polymerase (Invitrogen, Carlsbad, CA, USA). Touchdown PCR was performed under the following conditions: initial denaturation at 94 °C for 2 min, followed by 10 cycles of 94 °C for 30 s, 67–58 °C with a decrease of 1 °C per cycle for 30 s, and 72 °C for 2 min, followed by 25 cycles of 94 °C for 30 s, 58 °C for 30 s, and 72 °C for 2 min and finally 72 °C for 10 min for the final extension. The PCR products were examined by electrophoresis on 1% agarose gel and stained with ethidium bromide. DNA fragments were extracted from the ethidium bromide-stained gel, cloned into the pGEM-T Easy Vector System I (Promega, Madison, WI, USA), and transformed into the *Escherichia coli* strain DH5-α. The nucleotide sequences of the DNA fragments were determined using the Macrogen sequencing service (Seoul, Republic of Korea). The obtained sequences were verified by a homology search of the *FT* homologs in the database.

To obtain the complete sequence of *CnHd3a* genes, the 5′ and 3′ parts of the genes were amplified using PCR with specific primers designed from the previously obtained sequence of the conserved region and the *Heading date 3a* (*Hd3a)* sequence of oil palm (*Elaeis guineensis*) (XM_019849542).

**Figure 2:**
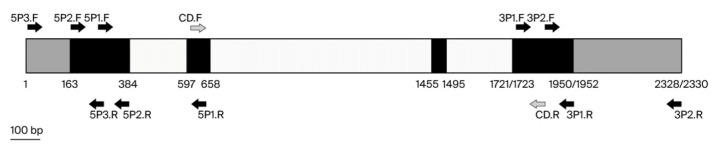
Position of primers designed for isolation of *CnHd3a* from coconut genome using PCR walking. Left and right arrows indicate forward and reverse primers, respectively. Primer pairs used were CD.F and CD.R, 5P1.F and 5P1.R, 5P2.F and 5P2.R, 5P3.F and 5P3.R, 3P1.F and 3P1.R, and 3P2.F and 3P2.R. Black, gray, and white boxes represent coding sequence (ORF), untranslated sequence, and intron, respectively. Numbers indicate position of nucleotide of *CnHd3a* genes in tall and dwarf coconuts.

The positions and series of primers used for PCR amplification are listed in Figure 2 and Table 1, respectively. The PCR components were the same as those previously mentioned. The series of DNA fragments was amplified using standard PCR under the same cycling conditions, except for the annealing temperature (Ta), which varied according to the specific primer pair used (see Table 1). The PCR cycle involved an initial denaturation at 94 °C for 2 min, followed by 35 cycles of 94 °C for 30 s, annealing at Ta for 30 s, extension at 72 °C for 30 s, and a final extension at 72 °C for 10 min. The amplified products were cloned into the pGEM-T Easy Vector System I (Promega, Madison, WI, USA) and sequenced. Overlapping DNA sequences were assembled using the CAP3 Sequence Assembly Program [46].

To verify the gene structure from genomic DNA, the cDNA of *CnHd3a* was isolated using the SuperScript™ III One-Step RT-PCR System with Platinum™ Taq DNA Polymerase (Invitrogen, Carlsbad, CA, USA) with a specific primer pair (5P3.F and 3P2.R ). One microgram of total RNA was used for the one-step RT-PCR, which involved incubation at 55 °C for 30 min, followed by 35 cycles at 94 °C for 15 s, 58 °C for 30 s, and 68 °C for 1 min and a final extension at 68 °C for 5 min. The resulting PCR product was cloned into the pGEM^®^-T Easy Vector (Promega, Madison, WI, USA) and sequenced by Macrogen, Inc. (Republic of Korea).

### 2.5. Sequence Analysis and Phylogenetic Tree

The nucleotide sequences were searched against the NCBI database using BLASTx and BLASTn programs [47]. The Splign program was used to detect splicing sites [48], and the ORF finder (https://www.ncbi.nlm.nih.gov/orffinder/, accessed on 20 March 2018) was used to determine the open reading frame sequences. Amino acid sequences were translated from the coding sequences using the ExPASy-translated tool (https://web.expasy.org/translate/, accessed on 20 March 2018). To identify homologs in other plants, amino acid sequences were searched against *FT* homologs submitted to GenBank using the BLASTp program [47]. Multiple sequence alignment was performed using ClustalW, and phylogenetic and molecular evolutionary analyses based on amino acid sequences were reconstructed using the Maximum Likelihood (ML) method in MEGA version 11 [49].

### 2.6. Expression Analysis of Different Alternative Transcripts Using RT-PCR

To study the expression patterns of different alternative splicing isoforms of *CnHd3a* transcripts, one-step RT-PCR was carried out on RNA templates from leaves and shoot apical meristems (SAMs) during vegetative growth (germinated and 6, 12, and 24 months of age) and reproductive growth (36 and 60 months of dwarf and tall, respectively), as well as the axial leaf of inflorescence and flowers (male and female) during the reproductive growth of dwarf coconut. Specific primers 5P2F and 3P2R (Table 1) were used to amplify multiple *CnHd3a* transcripts. The *Actin* gene was employed as a reference gene due to its constitutive expression. All reactions were run under the same conditions with 30 amplification cycles. RT-PCR products were separated on 4% UltraPure™ Agarose-1000 gels (Invitrogen, Carlsbad, CA, USA) and stained with ethidium bromide. The RT-PCR products were then extracted from the gel (Geneaid Biotech Ltd., New Taipei City, Taiwan), cloned into the pGEM^®^-T Easy Vector (Promega, Madison, WI, USA), and sequenced by Macrogen Inc., Seoul, Republic of Korea.

### 2.7. Gene Expression Analysis by Quantitative RT-PCR

To quantify the expression levels of *CnHd3a* transcripts in various tissues during development, quantitative RT-PCR was performed with specific primers (Table S1) and using 50 ng of cDNA as a template. The other PCR components and conditions were the same as mentioned previously, except for the annealing temperature and the number of cycles, which were 50 °C and 28 cycles, respectively. The PCR products were analyzed by agarose gel electrophoresis and stained with ethidium bromide. Band intensities were visualized and photographed using a Gel Doc imaging system (Bio-Rad Laboratories, Hercules, CA, USA) and quantified with Quantity One 1-D Analysis Software, version 4.6.8 software (Bio-Rad Laboratories, Hercules, CA, USA). Gene expression levels were determined using the relative quantification method, with coconut *Actin* (accession number: AM689520) serving as the reference gene.

## 3. Results

### 3.1. Cloning and Molecular Structure of CnHd3a from Dwarf and Tall Coconuts

To isolate the *FT* homolog gene that controls the flowering transition in coconut, the conserved region of the *FT* homolog gene, the PEBP (phosphatidylethanolamine-binding protein) domain, was amplified from dwarf and tall coconuts using touchdown PCR with degenerate primers designed from the conserved sequence of *FT* genes from other plants in GenBank, as described by Hou and Yang (2009) [45]. The amplified fragments of 1250 bp and 1248 bp were obtained from dwarf and tall coconuts, respectively. Their nucleotide sequences were highly homologous to those of the *FT* homolog gene, *Hd3a* (LOC105040685) from oil palm (*E. guineensis*). This indicated that both fragments were the central part of the *FT* homolog genes of dwarf and tall coconuts, which were named *CnHd3a (Heading date 3a)*. The 5′ and 3′ parts of the *CnHd3a* genes from both coconuts were amplified using walking PCR with gene-specific primers from the sequences of the obtained fragments and the sequence of the *Hd3a* mRNA of the oil palm (XM_019849542.2). The complete genomic sequences of *CnHd3a* were successfully isolated from dwarf and tall coconuts, with lengths of 2330 bp and 2328 bp, respectively. They were submitted to GenBank with accession numbers MH092984 and MN18152, respectively.

**Figure 3:**
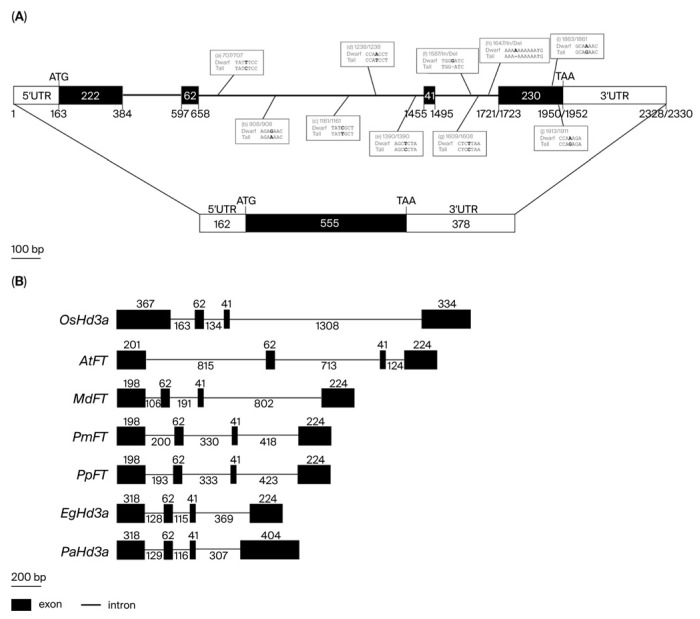
Genomic organization of *FT* homolog genes. (**A**) Gene organization of *CnHd3a* from dwarf and tall coconuts. Exon and intron regions are represented as boxes and lines, respectively; black boxes are ORF region; and white boxes are 5′ and 3′ untranslated regions. Numbers in boxes indicate lengths (base pairs) of regions. Numbers below exon boxes indicate position of DNA sequences of *CnHd3a* in tall and dwarf coconuts, respectively. Sequence variation between dwarf and tall coconuts is shown as open boxes with aligned DNA sequences; position and mutated nucleotides are shown as numbers and bold letters, respectively. (**B**) Genomic organization of *FT* homolog genes, *OsHd3a* in rice (*O. sativa*), *AtFt* in *Arabidopsis* (*A. thaliana*), *MdFT* in apple (*M. domestica*), *PmFT* in Japanese apricot (*Prunus mume*), *PpFT* in peach (*Prunus persica*), *EgHd3a* in African oil palm (*E. guineensis*), and *PdHd3a* in date palm (*Phonix dactylifera*). Accession numbers are as follows: LOC4340185, AF152096.1, DQ535887.1, AB444086.1, JF806623.1, KX242335.1, and NW_008262554.1, respectively. Exon and intron regions are represented by black boxes and lines, respectively. Number represents length (bp) of exons and introns.

**Figure 4:**
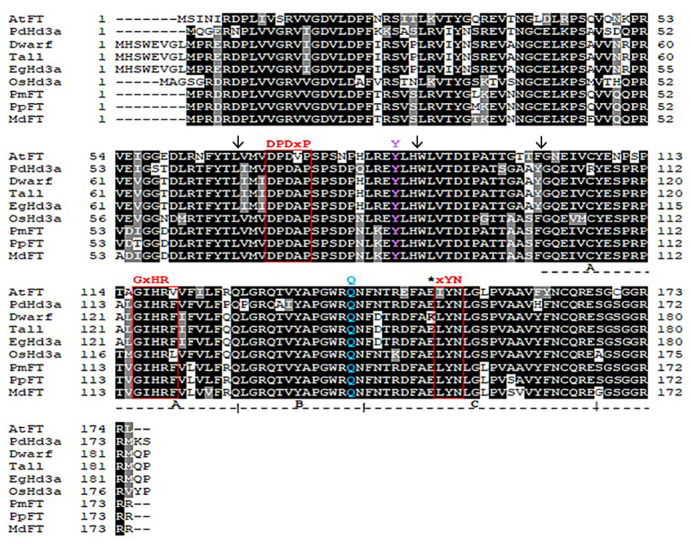
The alignment of the deduced amino acid sequences of *FT* homolog genes, including *OsHd3a* in rice (*O. sativa* ), *AtFt* in *Arabidopsis* (*A. thaliana*), *MdFT* in apple (*M. domestica*), *PmFT* in Japanese apricot (*P. mume*), *PpFT* in peach (*P. persica*), *EgHd3a* in African oil palm (*E. guineensis*), and *PdHd3a* in date palm (*Phonix dactylifera*). The accession numbers are as follows: NP_001408118.1, AAF03936.1, ABF84010.1, BAH82787.1, AE072030.1, AR0770145.1, and XP0087780096.1, respectively. Black and gray shading represent identical and similar amino acids, respectively. The exon-exon junctionpositions are marked by black vertical arrows above their sequences. The D-P-D-x-P, GxHR, and xYN motifs are indicated by red boxes. The conserved amino acids Tyr84 (Y) and Trp138 (Q) are indicated by Y (in pink) and Q (in blue) above their sequences. Dotted black lines with A, B, and C represent segments A, B, and C of the FT protein, respectively. The star indicates the amino acid substitution in the dwarf and tall coconuts.

The gene structure was analyzed using bioinformatics to detect intron/exon boundaries and open reading frames (ORFs). The results showed that the *CnHd3a* structure from both coconuts consisted of four exons (384, 62, 41, and 230 bp, respectively) and three introns (212, 796, and 227 (dwarf)/225 (tall) bp, respectively) (Figure 3A). The putative full-length mRNAs were 1095 bp, with a 555 bp open reading frame (ORF) encoding 184 amino acids, a 162 bp 5′ UTR, and a 378 bp 3′ UTR (Figure 3A). The variation in *CnHd3a* between dwarf and tall coconuts was investigated, and the results are shown in Figure 3A. Five SNPs (single-nucleotide polymorphisms) were found in intron 2. One SNP and two indels (insertions/deletions) were found in intron three. Additionally, two single-base substitutions were found in the coding sequence (CDS) at positions 1861 and 1931 in exon 4 (Figure 3A). Guanine (G) in the tall type was mutated to adenine (A) in the dwarf type at both positions, but it resulted in a change of only one amino acid from glutamine (GAA) in the tall coconut to lysine (AAA) in the dwarf coconut (Figure 3A and Figure 4).

The gene structure of *CnHd3a* was compared with that of *FT* genes from annual plants, including *OsHd3a* and *AtFT* from rice (*O. sativa*) and *Arabidopsis* (*A. thaliana*), respectively, and perennial plants, such as *MdFT*, *PmFT*, *PpFT*, *EgHd3a*, and *PdHd3a* from apple (*M. domestica*), Japanese apricot (*P. mume*), peach (*P. persica*), African oil palm (*E. guineensis*), and date palm (*P. dactylifera*), respectively. The *CnHd3a* gene structure resembled *FT* homolog genes from these plants with conserved exon numbers and positions; however, the introns varied in length. The lengths of exons 2 (62 bp) and 3 (41 bp) were conserved among the genes examined (Figure 3B).

### 3.2. Molecular Function of CnHd3a and Phylogenetic Tree

**Figure 5:**
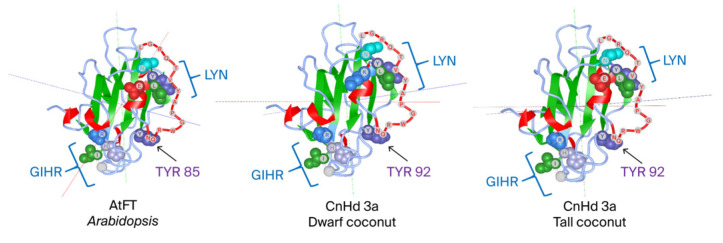
Predicted homology protein model of CnHd3a in dwarf and tall coconuts compared to AtFT in *Arabidopsis* (1AWF). The protein backbone is represented in ribbon format, with α-helices shown in red, β-sheets in green, and loop regions in light blue. External loop of segment B is represented in red with amino acid residues.The conserved GIHR and LYN sequences are annotated by individually highlighting each amino acid residue with distinct colors. In the GIHR motif, G is represented in light gray, I in green, H in light purple, and R in light blue. In the LYN motif, L is shown in green, Y in purple, and N in cyan-green.

**Figure 6:**
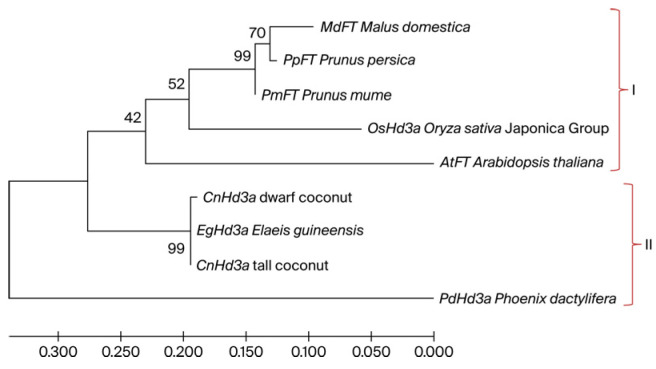
The phylogenetic tree of FT homologs, including OsHd3a in rice (*O.sativa*), AtFt in *Arabidopsis* (*A.thaliana*), MdFT in apple (*M. domestica*), PmFT in Japanese apricot (*P. mume*), *PpFT* in peach (*P. persica*), *EgHd3a* in African oil palm (*E. guineensis*), and PdHd3a in date palm (*P. dactylifera*). The accession numbers are as follows: NP_001408118.1, AAF03936.1, ABF84010.1,BAH8278.1, and 008780096.1, respectively.

To identify *CnHd3a* as a member of the *FT* homolog genes that function to promote flowering, the amino acid sequences of the CnHd3a protein were aligned with those of *FT homolog* proteins from *A. thaliana* (AAF03936.1), *O. sativa* (NP_001408118), *M. domestica* (ABF84010.1) , *P. persica* (AE072030.1) , *E. guineensis* (AR0770145.1) , and *P. dactylifera* XP008780096.1 , as shown in Figure 4. The CnHd3a protein displayed all the characteristic features of the FT-like protein subfamily [29], including the conserved key amino acids Tyr85 (Y) and Gln140 (Q) (Tyr91 and Gln141 in CnHd3a, respectively), the conserved motifs DPDxP and GxHR, and the highly conserved amino acid sequences LGRQTVYAPGWRQN and LYN, corresponding to the binding regions of FT with FD and the 14-3-3 protein [22,50] found in exon IV (Figure 4). In addition, the putative amino acid sequences of CnHd3a were used to construct tertiary structure models, which showed homology to the FT protein in *Arabidopsis* and the Heading 3a protein in rice, both of which function to promote the flowering transition (Figure 5). Taken together, these data demonstrate that CnHd3a clusters robustly within the FT-like subfamily and shares all conserved motifs characteristic of florigen proteins. To evaluate the molecular evolution of *CnHd3a* compared to that of its *FT* homologs, a phylogenetic tree was constructed based on their amino acid sequences. The results revealed that palm plants were separated from the others. *CnHd3a* from both coconuts was grouped with Palmae, and coconuts showed the closest relationship with the oil palm *E. guineensis* as a monophyletic branch (Figure 6).

To further investigate whether multiple *CnHd3a* genes exist within the coconut genome, we performed a genome-wide search for *PEBP* gene family members using the latest genome assemblies [9,51]. A total of 10 *PEBP* gene loci were identified. Among these, only one locus shared high sequence identity (>98%) with the full-length *CnHd3a* cDNA obtained from both the tall and dwarf coconut varieties. To determine the evolutionary relationship of CnHd3a between the coconut PEBP members, we conducted a phylogenetic analysis based on amino acid sequences. The predicted protein sequences were aligned using MUSCLE, and a Maximum Likelihood (ML) tree was constructed with 1000 bootstrap replicates. The resulting tree (Supplementary Figure S3) revealed that CnHd3a clustered within the FT-like subclade, clearly separated from the TFL1-like and MFT-like subfamilies. This placement was supported by strong bootstrap support (96%), indicating its close relationship with known floral inducers such as *Arabidopsis* FT and rice Hd3a.

### 3.3. Spatio-Temporal Expression Patterns of CnHd3a

**Figure 7:**
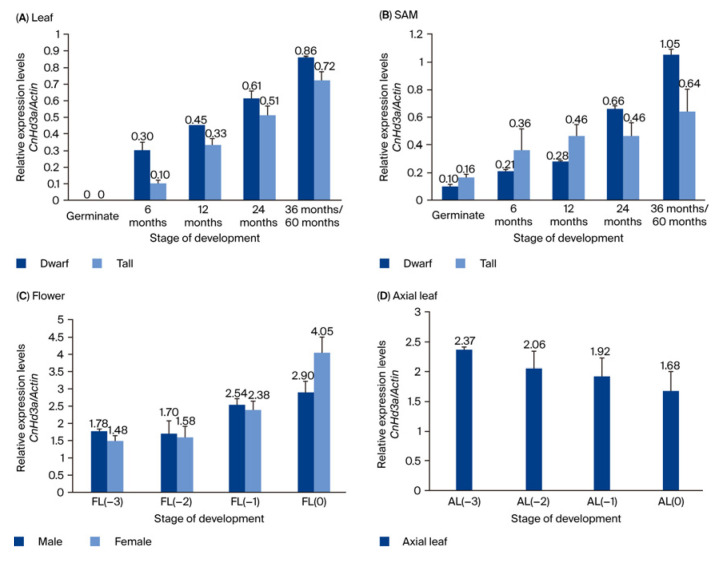
The expression profiles of *CnHd3a* within various organs in different developmental stages: (**A**) leaf; (**B**) SAM; (**C**) flower; (**D**) axial leaf.

Figure 7 shows the expression patterns of *CnHd3a* genes in dwarf and tall coconuts during growth and development. The results showed that *CnHd3a* was expressed in leaves and the SAM during the floral transition in both types. There were no significant differences in the trends in expression levels between the two types, with both showing an increasing trend and reaching the highest expression level during the 36–60 months period, at the reproductive stage. These findings suggest that *CnHd3a* may play a role in regulating the floral transition in coconuts, but further investigation is needed to fully understand its function. At the reproductive stage, *CnH3a* was found to be expressed in male and female flower and axial leaves. Its expression level tends to increase during flower development, peaking at the open inflorescence stage (stage 0) (Figure 7C); however, no significant differences were observed among the axial leaves. This implies a potential role for *CnHd3a* in the regulation of flower development.

### 3.4. The Alternative Splicing of the CnHd3a Gene

During the isolation of *CnHd3a* cDNA from leaves and the SAM at the reproductive stage using one-step RT-PCR with specific primers (5P2F and 3P2R), multiple PCR products were obtained. We hypothesized that there are multiple isoforms of *CnHd3a* transcripts. Therefore, the amplified fragments were cloned and sequenced. The sequencing results revealed that they were transcribed from *CnHd3a*, suggesting that *CnHd3a* functions to control the flowering transition through alternative splicing forms.

**Figure 8:**
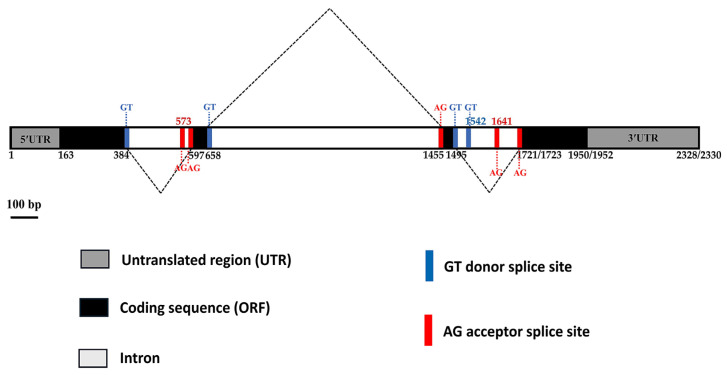
Potential alternative splicing sites in each intron of *CnHd3a*. Blue and red vertical lines represent the donor and acceptor sites, respectively.

**Figure 9:**
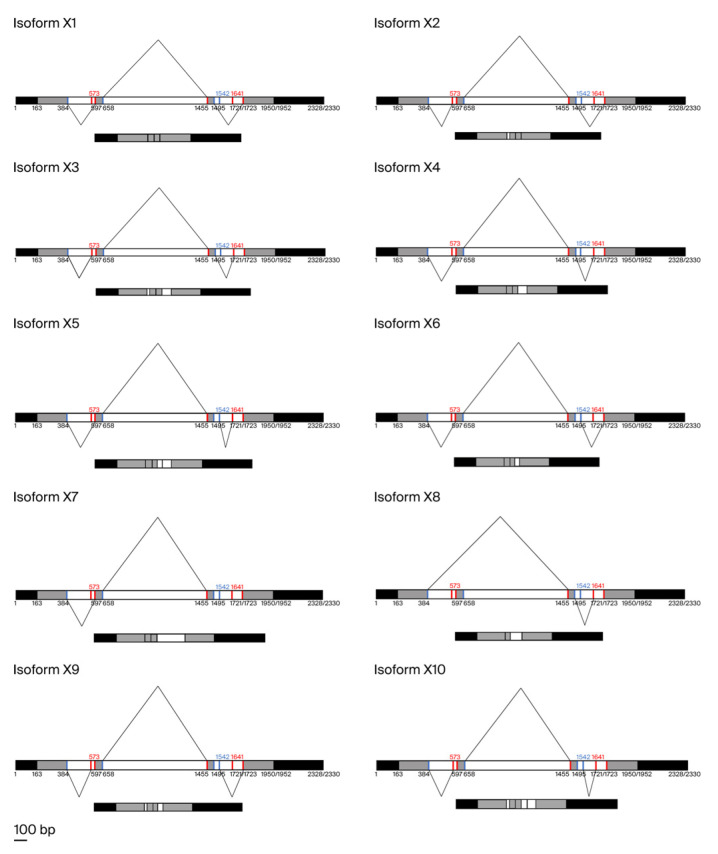
Putative alternative splicing of *CnHd3a* in coconuts.

To further investigate the alternative isoforms of *CnHd3a* transcripts, the sequences of the three introns were analyzed to detect potential splicing sites, as shown in Figure 8. The first intron contained one potential donor and two potential acceptors. The second intron had one potential donor and one potential acceptor. The third intron contained two potential donors and two potential acceptors. Ten splice variants could potentially be generated based on alternative acceptor and donor sites. The conserved isoform was named X1, and the others were named X2–X10, as shown in Figure 9.

**Figure 10:**
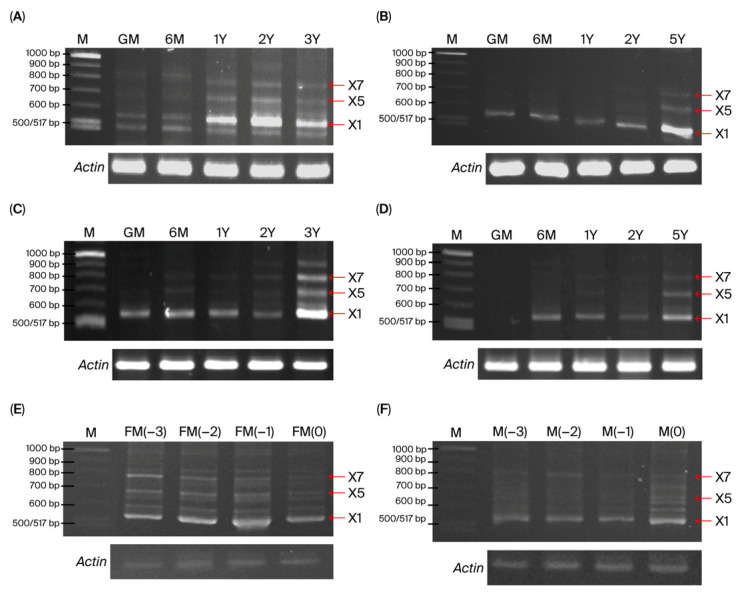
Temporal and spatial expression of alternative isoforms of *CnHd3a* in leaves (**A**,**B**) and SAM (**C**,**D**) of dwarf and tall coconuts, respectively, in female (**E**) and male (**F**) flowers of dwarf coconuts. *Actin* was used as internal reference gene. Although *Actin* bands appear intense, same RT-PCR conditions and cycle numbers were applied to all samples, and consistent signal across lanes supports its use as comparative control. PCR products were separated on 4% UltraPure™ Agarose 1000 (Invitrogen, Thermo Fisher Scientific, USA) to allow for visualization of subtle size differences among splice variants.

RT-PCR was performed using specific primers to monitor the expression of distinct *CnHd3a* transcript isoforms in different tissues and developmental stages. The PCR products of the various transcripts were separated on a high-resolution agarose gel. The conserved isoform (X1, 555 bp) was ubiquitously expressed in all investigated tissues (leaves, SAM, and flowers), as shown in Figure 10. In the SAM and leaves, the expression levels significantly increased during the transition to the reproductive stage. In contrast, isoforms X5 (682 bp in tall; 683 bp in dwarf) and X7 (780 bp in tall; 782 bp in dwarf) were detected only during the reproductive stage (Figure 10). This pattern was observed in both types of coconut, suggesting that the expression level and combination of alternative *CnHd3a* isoforms were associated with controlling the flowering transition in coconut. Interestingly, the complex isoform was expressed early in 1-year-old leaves in dwarf coconut, while delayed expression (5-year age) was observed in tall coconut. This suggested that alternative isoforms were involved in shortening the vegetative growth phase of the dwarf coconut. In addition to the expected isoform X1, a faint band smaller than X1 was detected exclusively in the leaf tissue of dwarf coconut (Figure 10A). This band may represent a novel splice variant. However, further sequencing analysis is required to determine its precise identity. In lane 3Y of Figure 10C, we observed an additional band larger than the expected X7 isoform. This band may correspond to an incompletely spliced transcript or an alternatively spliced variant containing retained intronic sequences. While this fragment was reproducibly detected, its exact sequence and identity were not characterized in this study.

During the reproductive stage, isoform X1 is predominantly expressed in male and female flowers and axial leaves. Its expression level gradually increased during flower development (Figure 10). This result suggests that isoform X1 is also involved in flower development.

To investigate the functional relevance of alternative splicing in CnHd3a, we compared the amino acid sequences and predicted tertiary structures of three major splice variants: X1, X5, and X7. Sequence alignment showed that all isoforms conserved the hallmark “DPDxP” motif and the critical Tyr-92 residue (analogous to Tyr-85 in *Arabidopsis* FT) located in exon 2 (Figure 5; Supplementary Figure S4 ). These residues are essential for the interaction between FT proteins and 14-3-3 scaffold proteins within the florigen activation complex (FAC), a mechanism conserved across angiosperms [52,53]. However, structural modeling revealed clear differences among the isoforms (Supplementary Figure S4). Isoform X1 retained the complete external loop structure and β-sheet elements associated with FT protein function, including a surface-exposed Tyr-92 positioned optimally for 14-3-3 interaction, which is required for FAC assembly and the transcriptional activation of flowering genes [54]. In contrast, isoforms X5 and X7, while preserving the core FT motifs in sequence, lacked this critical external loop architecture. The absence of the loop may impair their ability to bind FT-interacting proteins such as FD, thus diminishing their activity in flowering induction [55].

## 4. Discussion

The molecular characterization of *CnHd3a* and the spatial expression of its alternative splicing forms revealed crucial insights into the flowering transition and flower development in dwarf and tall coconut palm (*C. nucifera*). We confirmed and characterized *CnHd3a* as an *FT-like* gene that functions in controlling the flowering transition in coconuts and further examined the isoforms generated through alternative splicing, their expression patterns in various tissues, and their association with the flowering transition and flower development.

In our study, *CnHd3a* was successfully isolated from both dwarf and tall coconuts, and its genomic sequence showed high homology with the *FT homolog gene heading date 3a-like* (LOC105040685) from oil palm (*E. guineensis*). The gene structure of *CnHd3a* consisted of four exons and three introns, with a few SNPs and indels found between the dwarf and tall coconut types. Interestingly, the gene structure comparison of *CnHd3a* with *FT* genes from other annual and perennial plants revealed conserved exon numbers and positions, despite differences in intron lengths. The presence of conserved amino acid sequences corresponding to the binding regions of FT with FD and the 14-3-3 protein further confirmed that *CnHd3a* is a member of the FT-like subfamily that controls the flowering transition in coconuts. Phylogenetic analysis based on amino acid sequences revealed that the CnHd3a of both coconuts grouped with Palmae, with tall coconuts showing the closest relationship to oil palm (*E. guineensis)*.

*CnHd3a* was expressed in leaves, the shoot apical meristem (SAM), and flowers, indicating that it may play a significant role in regulating the floral transition process in coconuts. Interestingly, the expression levels of *CnHd3a* were found to increase gradually and reached their peak during the 36–60 months period, with no significant differences observed between the two types of coconuts. During the isolation of *CnHd3a* cDNA from leaves and the SAM during the reproductive stage, multiple PCR products were found, suggesting the existence of multiple isoforms of *CnHd3a* transcripts. A further analysis of intron sequences revealed potential splicing sites, generating ten splice variants through alternative acceptor and donor sites. Alternative splicing is a crucial mechanism contributing to gene regulation and plant proteome diversity. In most flowering plants, the introns present in genes are spliced from pre-mRNA before mature mRNA is formed. Variations in splice site junctions between introns and exons lead to the production of multiple mRNA transcripts or isoforms from a single gene [56]. The *FT* gene, which plays a central role in the floral transition from vegetative to reproductive growth in *A. thaliana*, has also been shown to undergo alternative splicing in other species, with functional implications. In *Chrysanthemum morifolium,* for example, alternatively spliced isoforms of *CmFTL1* exhibit varying capacities to induce flowering depending on exon composition and the floral developmental stage. Specifically, isoforms that retain exon 2 are capable of fully rescuing the ft-10 mutant phenotype in *Arabidopsis*, whereas those lacking exon 2 fail to do so [57]. Similarly, in temperate grasses such as *Brachypodium distachyon*, barley, and wheat, an *FT* splice variant lacking the region required for forming the florigen activation complex (FAC) acts as a dominant-negative regulator of flowering. This highlights that the retention of key motifs—particularly within exon 2—is essential for FT protein activity [58]. These findings support the notion that the structural integrity of FT-like proteins, maintained through conserved splicing patterns, is necessary for their florigenic function. In the case of coconuts, Xia et al. (2020) [41] detected the alternative splicing of a *CnFT* gene between tall and dwarf coconuts. They found only one transcript in dwarf coconut, which lacked six nucleotides, and inferred that it contributed to the early flowering phenotype in coconut positively. Notably, they detected another isoform of *CnFT* in 8 out of 11 tall coconuts. They argued that the remaining three might potentially result from open pollination or heterozygosity resulting from cross-pollination with dwarf coconut varieties. The discovery of alternative splice variants of *CnFT* in tall and dwarf coconuts provides a potential explanation for the differences in flowering time between these two coconut types.

In our study, the conserved isoform (X1) was ubiquitously expressed in all stages of the investigated tissues, while other isoforms, such as X5 and X7, were found only at the reproductive stage. The expression patterns of the distinct isoforms of *CnHd3a* transcripts in different tissues and stages of development revealed that the conserved isoform X1 was highly increased during the transition to the reproductive stage. Interestingly, complex isoforms were expressed earlier in dwarf coconut leaves than in tall coconut leaves, suggesting their involvement in shortening the vegetative growth phase of dwarf coconuts. These observations suggest that the differential expressions and alternative splicing of *CnHd3a* may contribute to the fine-tuning of floral transition timing in coconut. The early appearance of isoforms X5 and X7 in dwarf coconut coincides with the early flowering phenotype commonly observed in this variety, implying a regulatory mechanism involving specific splicing variants. Given that both X5 and X7 retain the essential “DPDxP” motif and Tyr-92 residue within exon 2—key determinants of FT function—these isoforms may still possess partial florigenic activity. Such a splicing-mediated modulation of FT homolog activity has been documented in other species, including *Chrysanthemum morifolium* and cereals, where exon-skipped variants resulted in reduced or abolished florigen function [57,58]. Therefore, the presence and timing of the expression of these isoforms may serve as an intrinsic mechanism for controlling flowering time in different coconut varieties. Moreover, during the reproductive stage, isoform X1 was predominantly expressed in flowers, male and female flowers, and axial leaves, and its expression level gradually increased with flower development (Figure 7 and Figure 10). These findings indicated that isoform X1 was involved in flower development. Although flower samples were collected only from dwarf coconuts due to practical limitations, we hypothesize that the regulatory mechanism of *CnHd3a* isoform X1 during floral development is likely conserved between tall and dwarf coconut varieties. This assumption is supported by the evolutionary conservation of *FT-like* genes in the floral transition and development across diverse plant species.

Our phylogenetic analysis of the coconut *PEBP* gene family further supports the identification of *CnHd3a* as a bona fide *FT-like* gene. Although ten *PEBP* loci were detected in the coconut genome, only one showed high similarity to the cloned *CnHd3a* transcript. The ML tree based on amino acid sequences confirmed that CnHd3a belongs to the FT-like clade, with strong statistical support, distinguishing it from TFL1/CET/SP-like and MFT-like paralogs. These results suggest that the alternative splicing isoforms we observed are derived from a single functional *FT-like* gene rather than multiple gene copies or recent duplications. This reinforces the hypothesis that *CnHd3a* plays a conserved role in flowering regulation in coconuts, like *FT* homologs in other species.

The molecular characterization of *CnHd3a* and the spatial expression of its alternative splicing forms have shed light on the intricate mechanisms controlling the flowering transition and flower development in the coconut palm. This study provides a solid foundation for future research aimed at elucidating the precise functions and interactions of *CnHd3a* in regulating coconut palm growth and development. The identification of distinct isoforms and their expression patterns in different tissues and developmental stages suggests that the regulation of the flowering transition and flower development in the coconut palm is a complex process involving multiple isoforms of *CnHd3a*. Furthermore, the observation that complex isoforms are expressed earlier in dwarf coconuts than in tall coconuts indicates a possible role for these isoforms in determining the length of the vegetative growth phase. This finding could have implications for coconut breeding programs aimed at developing varieties with shorter vegetative growth phases and earlier flowering, which could lead to increased productivity.

Further research should focus on functionally characterizing each isoform through functional assays, such as overexpression or gene knockout experiments, to determine their specific roles in the flowering process. Additionally, exploring the upstream regulatory pathways and downstream target genes of *CnHd3a* and its isoforms would provide a more comprehensive understanding of the molecular networks governing the flowering transition and flower development in the coconut palm.

It would also be valuable to investigate the potential interactions between *CnHd3a* isoforms and other key flowering regulators, such as *CONSTANS (CO)* and *FLOWER-ING LOCUS T (FT)*, as well as the possible involvement of epigenetic regulation, hormonal signaling, and environmental cues in the modulation of *CnHd3a* expression and function. Moreover, a comparative analysis of *CnHd3a* and its isoforms in different coconut varieties with varying flowering habits could provide valuable insights into the genetic basis of flowering time variation in the coconut palm. This information can be used to develop molecular markers for the selection of desirable flowering traits in breeding programs, ultimately contributing to the improvement of coconut palm productivity and adaptability to changing environmental conditions.

## 5. Conclusions

This study presents the molecular and functional characterization of *CnHd3a*, a coconut *FT* homolog gene, and its alternatively spliced isoforms in both dwarf and tall varieties of Cocos nucifera. The gene structure consists of four exons and three introns, consistent with other *FT/TFL1* family members. Sequence and phylogenetic analyses confirmed its classification as an *FT-like* gene, possessing conserved motifs and residues required for floral induction. Expression profiling revealed that *CnHd3a* is predominantly expressed in leaves and shoot apical meristems during the reproductive transition, with earlier peak expression in dwarf palms. Alternative splicing produced ten isoforms (X1–X10); notably, isoforms X5 and X7 were detected only during flowering stages, appearing earlier in dwarf palms, suggesting a role in shortening the juvenile phase. These results highlight *CnHd3a*, particularly its complex isoforms, as a key regulator of flowering in coconut. The discovery of developmentally and varietally specific splice forms provides valuable targets for molecular breeding strategies to accelerate flowering and improve yield. Further functional studies are recommended to clarify the specific roles of individual isoforms in flowering regulation.

## Figures and Tables

**Table 1:** Name and sequence of primers used to clone full-length *CnHd3a* gene.

Primer Name	Nucleotide Sequence (5′–3′)	Tm (°C)	Ta (°C)
CD.F	ATGGTGGATCCNGAYGYNCCNAGYCC	59.0	55–64
CD.R	GTGYTGAAGTTCTGRCGCCACCCNGG	62.0	
5P2.F	ATGCACTCTTGGGAGGTAGGA	61.3	58
5P2.R	AAGGGTGTAGAAGGTCCTGAGG	64.0	
3P1.F	GGTCAGGAGATTGTGTGCTATGAGAGTCC	71.9	64
3P1.R	TTAAGGTTGCATCCTTCTCCCGCC	67.0	
5P3.F	GCCTAAAGTCTGTGTGCCAAG	61.3	55
5P3.R	TGCCTCCAACCTCAACCCTAG	63.3	
5P1.F	CCCCTCAGGGTGATCTACAA	60.5	54
5P1.R	AACTCTTCAGCGGGTGCTTA	58.4	
3P2.F	CGGTCGCAGCAGTCTATTTT	58.4	50
3P2.R	TACCCGAAAAGTTCACTAATT	53.5	

## Data Availability

The data is available upon request from the corresponding author.

## Supplementary Materials

The following supporting information can be downloaded at https://www.mdpi.com/article/10.3390/genes16060718/s1. Table S1. Primer for expression analysis of *CnHd3a* gene; Figure S1. The amplified conserved regions of PEBP gene family from dwarf (D) and tall (T) coconuts. The size of DNA fragments are indicated, 1250 and 1248 bp from dwarf and tall respectively; Figure S2. The comparison of genomic DNA sequence of *CnHd3a* from tall and dwarf coconuts ( CLUSTAL O (1.2.4) multiple sequence alignment). The grey colors indicate intron regions of conserved isoform. GT-AG splicing motifs are indicated by blue and red colors, respectively. *GT-AG* italic fronts indicate alternative donor and acceptor sites, respectively; Figure S3. Phylogenetic tree of 10 predicted PEBP family members from coconut genome and CnHd3a of dwarf and tall coconuts. The tree was constructed using the ML method with 1000 bootstrap replicates. Bootstrap values are indicated at the nodes. Evolutionary analyses were conducted in MEGA11 [50]; Figure S4. Multiple sequence alignment (A) and structural comparison of CnHd3a isoforms (X1, X5, and X7) from tall and dwarf coconut (B). Conserved motifs (DPDxP, GxHR, and xYN) and the critical residue Tyr-92 (Y) are annotated on the amino acid sequence, with Y highlighted in yellow. Black arrows mark the exon–exon junctions. Predicted 3D structures show the GIHR and LYN motifs labeled in blue, with secondary structures displayed as red α-helices, green β-sheets, and light blue loops. The critical residue Tyr-92 (Y) is also represented as an individual purple amino acid in the 3D structure. The external loop of segment B, highlighted in red with labeled amino acids, is present only in isoform X1 and absent in X5 and X7.

## Abbreviations

The following abbreviations are used in this manuscript:

SAM	Shoot apical meristem
FT	Flowering locus T
Hd3a	Heading date 3A
