# Supplementary material for: Molecular Characterization of CnHd3a and Spatial Expression of Its Alternative Splicing Forms Associated with Flowering Transition and Flower Development in Coconut Palm (Cocos nucifera L.)
Source: Genes (Basel). 2025 Jun 18;16(6):718. doi: 10.3390/genes16060718 (PMC12193392; doi:10.3390/genes16060718)
Supplement: Supplementary file 1 [file genes-16-00718-s001.zip › genes-3653259-supplementary.pdf]

Table S1. Primer for expression analysis of *CnHd3a*

Primer name	nucleotide sequence (5′→ 3′)	Tm (°C)	Ta (°C)
Hd3a_3'UTR_F	CGGTCGCAGCAGTCTATTTT	58.4	50
Hd3a_3'UTR_R	TACCCGAAAAGTTCACTAATT	53.5	
Cocos_Act_F	GGCAGAACGGGAAATTGTAA	55.2	50
Cocos_Act_R	CCAATTAAGGATGGCTGGAA	55.2	

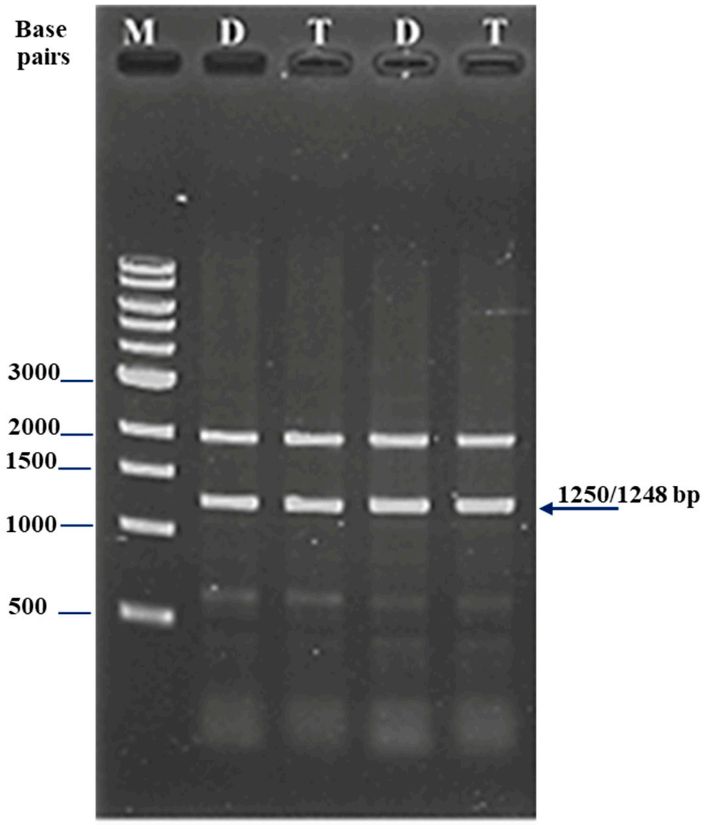

Figure S1. The amplified conserved regions of PEBP gene family from dwarf (D) and tall (T) coconuts. The size of DNA fragments are indicated, 1250 and 1248 bp from dwarf and tall respectively.

CLUSTAL O(1.2.4) multiple sequence alignment

```
Dwarf      GCCTAAAGTCTGTGTGCCAAGGTTGCTCTCATTAGCCTTCTAAACAAAAGCATCAAAGTC   60
Tall       GCCTAAAGTCTGTGTGCCAAGGTTGCTCTCATTAGCCTTCTAAACAAAAGCATCAAAGTC   60
           ************************************************************

Dwarf      CCCACCTGTTCCCCTTTGTTTTAATTACCCCATCTTCGATCTCATACTTCTCTGCCAGCA   120
Tall       CCCACCTGTTCCCCTTTGTTTTAATTACCCCATCTTCGATCTCATACTTCTCTGCCAGCA   120
           ************************************************************

Dwarf      GCCTGTCTTACCATAAGATCCTAATTCTTTTTTCCTCACCAGATGCACTCTTGGGAGGTA   180
Tall       GCCTGTCTTACCATAAGATCCTAATTCTTTTTTCCTCACCAGATGCACTCTTGGGAGGTA   180
           ************************************************************

Dwarf      GGACTCATGCCAAGAGAAAGGGATCCTTTGGTTGTGGGCAGGGTGATAGGAGATGTGTTG   240
Tall       GGACTCATGCCAAGAGAAAGGGATCCTTTGGTTGTGGGCAGGGTGATAGGAGATGTGTTG   240
           ************************************************************

Dwarf      GATCCATTCATAAGAAGTGTACCCCTCAGGGTGATCTACAATTCGAGGGAGGTGGCCAAT   300
Tall       GATCCATTCATAAGAAGTGTACCCCTCAGGGTGATCTACAATTCGAGGGAGGTGGCCAAT   300
           ************************************************************

Dwarf      GGATGTGAGCTTAAACCCTCAGCTGTCGTTAACCGACCTAGGGTTGAGGTTGGAGGCACT   360
Tall       GGATGTGAGCTTAAACCCTCAGCTGTCGTTAACCGACCTAGGGTTGAGGTTGGAGGCACT   360
           ************************************************************

Dwarf      GACCTCAGGACCTTCTACACCCTTGTAAGCCCATCTCTCTCTCTCTCTCTCTCCCCCTAG   420
Tall       GACCTCAGGACCTTCTACACCCTTGTAAGCCCATCTCTCTCTCTCTCTCTCTCCCCCTAG   420
           ************************************************************

Dwarf      ATGCGTATCTTCTTGGGGGCTTACCATTCGATCTCCTTGCAAAAGTGCTACTTTTGAATT   480
Tall       ATGCGTATCTTCTTGGGGGCTTACCATTCGATCTCCTTGCAAAAGTGCTACTTTTGAATT   480
           ************************************************************

Dwarf      TGAATTTGAATTTGCATTTGGCGAAGCCCAGTCTCTTTTTTTTCTTTTTCTTTTTTTCTTCT   540
Tall       TGAATTTGAATTTGCATTTGGCGAAGCCCAGTCTCTTTTTTTTCTTTTTCTTTTTTTCTTCT   540
           ************************************************************

Dwarf      TTTCTGACATGGTGATATTTGCTAGTTCTTAGGGTGTTCTTAACGATGTCTTGCAGATTA   600
Tall       TTTCTGACATGGTGATATTTGCTAGTTCTTAGGGTGTTCTTAACGATGTCTTGCAGATTA   600
           ************************************************************

Dwarf      TGATAGACCCGGATGCTCCAAGTCCAAGTGACCCACACCTCAGGGAGTATTTGCACTGGT   660
Tall       TGATAGACCCGGATGCTCCAAGTCCAAGTGACCCACACCTCAGGGAGTATTTGCACTGGT   660
           ************************************************************
                                                         707
Dwarf      AAGCACCCGCTGAAGAGTTGTACCCATCGGGAAGCCATGAGCATATTTCCTTTTGCATTA   720
Tall       AAGCACCCGCTGAAGAGTTGTACCCATCGGGAAGCCATGAGCATATCTCCTTTTGCATTA   720
           ********************************************** *************

Dwarf      CTTTGAGGATTTATTTTTGCTATATATTGGGCCGGACTTCACTCGCATGCTATTGAGAGA   780
Tall       CTTTGAGGATTTATTTTTGCTATATATTGGGCCGGACTTCACTCGCATGCTATTGAGAGA   780
           ************************************************************

Dwarf      AGAACAAGGAAGAGAAGAGATTGCGGAACAAAACTAGCTCCACAAATGTTTTATTAATCA   840
Tall       AGAACAAGGAAGAGAAGAGATTGCGGAACAAAACTAGCTCCACAAATGTTTTATTAATCA   840
           ************************************************************

Dwarf      AGCAAAATATATAATCTAGTGCAAACTCAACCCTAGGTCTAGCCTTCCAAATCCGACTGA   900
Tall       AGCAAAATATATAATCTAGTGCAAACTCAACCCTAGGTCTAGCCTTCCAAATCCGACTGA   900
           ************************************************************
                  908
Dwarf      AAATAGAGAACCTGGCCCAAAAATAAAAAACAAATTTTCTAGGACTACTCGACTGCAAAA   960
Tall       AAATAGAAAACCTGGCCCAAAAATAAAAAACAAATTTTCTAGGACTACTCGACTGCAAAA   960
           ******* ****************************************************
```

```
Dwarf       TTCGAAAATTAGGCCGAAACCTAGTACTGAACCTGCTCCTGCGGGCTTTGGTACAGTTCA  1020
Tall        TTCGAAAATTAGGCCGAAACCTAGTACTGAACCTGCTCCTGCGGGCTTTGGTACAGTTCA  1020
            ************************************************************

Dwarf       TCTAACCTGCAAATATGCCTTCAGTTTCTATAGTTTAGTTCTAACAGAAACTTGAGTTCT  1080
Tall        TCTAACCTGCAAATATGCCTTCAGTTTCTATAGTTTAGTTCTAACAGAAACTTGAGTTCT  1080
            ************************************************************

Dwarf       TTGGATATATGAAAAGCTCGACAACCCAATTGCCTAAAAGCCAATTCTGATCCAAAGCCC  1140
Tall        TTGGATATATGAAAAGCTCGACAACCCAATTGCCTAAAAGCCAATTCTGATCCAAAGCCC  1140
            ************************************************************
                                1161
Dwarf       GGTATACAATCCAGGCATATCGCTTGGCAAAATCAAACTTTAATCATGTATTTTTCATCT  1200
Tall        GGTATACAATCCAGGCATATTGCTTGGCAAAATCAAACTTTAATCATGTATTTTTCATCT  1200
            ******************** ***************************************
                                                 1238
Dwarf       TAACTAGGCTGTTTATATGCTTCAAACCATTGTACCAACCTGCACTTGTCAGAGAAAAGT  1260
Tall        TAACTAGGCTGTTTATATGCTTCAAACCATTGTACCATCCTGCACTTGTCAGAGAAAAGT  1260
            ************************************* **********************

Dwarf       TCGCATTGTCTCCATGATGCAAATGCACAGGCACCAAAAACATTGCAGAAACGTCACTAA  1320
Tall        TCGCATTGTCTCCATGATGCAAATGCACAGGCACCAAAAACATTGCAGAAACGTCACTAA  1320
            ************************************************************

Dwarf       ATCCCTAACTTATGAAATTGCACTTCCAATCTCTCAAAGACTTCCATGGATCCAAAGCTA  1380
Tall        ATCCCTAACTTATGAAATTGCACTTCCAATCTCTCAAAGACTTCCATGGATCCAAAGCTA  1380
            ************************************************************
                   1390
Dwarf       ATAATCAGCTCTACCCTCGAGCTTTTCTAGTAAAGTTCTTGCAATACGCACTAATGTGAT  1440
Tall        ATAATCAGCCCTACCCTCGAGCTTTTCTAGTAAAGTTCTTGCAATACGCACTAATGTGAT  1440
            ********* **************************************************

Dwarf       CTTTGCTGCTGTAGGTTGGTCACGGATATCCCGGCAACGACTGGAGCTGCCTACGGTAAG  1500
Tall        CTTTGCTGCTGTAGGTTGGTCACGGATATCCCGGCAACGACTGGAGCTGCCTACGGTAAG  1500
            ************************************************************

Dwarf       ATCACAAGAACCAAAAACGATAAGTCCTCCCAAGTGGATCAGGTTATCATTTAGCTTCTA  1560
Tall        ATCACAAGAACCAAAAACGATAAGTCCTCCCAAGTGGATCAGGTTATCATTTAGCTTCTA  1560
            ************************************************************
                                   1587/1586             1609/1608
Dwarf       GCTGCCTGTGTTTCCGACAGTACTGGGATCAAAATGCCTCATCTACTCTTAATTTAATCA  1620
Tall        GCTGCCTGTGTTTCCGACAGTACTGG-ATCAAAATGCCTCATCTACTCCTAATTTAATCA  1619
            ************************** ********************* ***********
                                   1647/1645 (In/Del)
Dwarf       CCTACGTTTCAACATTTACAGCCAAAAAAAAAAATGTCGTCGCAGAAGAATGATTGGCAGG  1680
Tall        CCTACGTTTCAACATTTACAGCCAAA-AAAAAATGTCGTCGCAGAAGAATGATTGGCAGG  1678
            ************************** *********************************

Dwarf       CCTGATCATCACTATGTCTCATAATTTTATACCTGGCTTCAGGTCAGGAGATTGTGTGCT  1740
Tall        CCTGATCATCACTATGTCTCATAATTTTATACCTGGCTTCAGGTCAGGAGATTGTGTGCT  1738
            ************************************************************

Dwarf       ATGAGAGTCCACGGCCGGCGCTTGGCATCCACCGGTTCATCTTTGTGCTGTTCCAGCAGC  1800
Tall        ATGAGAGTCCACGGCCGGCGCTTGGCATCCACCGGTTCATCTTTGTGCTGTTCCAGCAGC  1798
            ************************************************************

Dwarf       TTGGGCGGCAGACAGTGTATGCCCCTGGGTGGCGCCAAAATTTCGACACCCGGGACTTTG  1860
Tall        TTGGGCGGCAGACAGTGTATGCCCCTGGGTGGCGCCAAAATTTCGACACCCGGGACTTTG  1858
            ************************************************************
            1863/1861                                        1915/1913
Dwarf       CAAAACTCTACAACCTCGGATCACCAGTCGCAGCAGTCTATTTTAACTGCCAAAGAGAGT  1920
Tall        CAGAACTCTACAACCTCGGATCACCAGTCGCAGCAGTCTATTTTAACTGCCAGAGAGAGT  1918
            ** ************************************************* *******

Dwarf       CGGGCTCCGGCGGGAGAAGGATGCAACCTTAAACTATGGCTGATATTGTGAAATTGGGAG  1980
Tall        CGGGCTCCGGCGGGAGAAGGATGCAACCTTAAACTATGGCTGATATTGTGAAATTGGGAG  1978
            ************************************************************
```

```
Dwarf       CTATAGCTTGCACAGTGCCACCTCTCCTATTTTCTTGCAACGGGGATTTACATAGCATTA   2040
Tall        CTATAGCTTGCACAGTGCCACCTCTCCTATTTTCTTGCAACGGGGATTTACATAGCATTA   2038
            ************************************************************

Dwarf       TTGGGGACTTCACAGTGTCTAAAGAAAAATAATGGCTCCGGAAGTGAAGCTAGTCGAAAA   2100
Tall        TTGGGGACTTCACAGTGTCTAAAGAAAAATAATGGCTCCGGAAGTGAAGCTAGTCGAAAA   2098
            ************************************************************

Dwarf       GTGGAGATAGCTGGACAATATTCGGGGTTGAGATAGTAGCAGATAGCAGTAGAAGCTTTG   2160
Tall        GTGGAGATAGCTGGACAATATTCGGGGTTGAGATAGTAGCAGATAGCAGTAGAAGCTTTG   2158
            ************************************************************

Dwarf       GATTTCATGAAAAAGCTCAATAGATTGAACACATTAGCCCTTTTTTTCATGATTGAAAAT   2220
Tall        GATTTCATGAAAAAGCTCAATAGATTGAACACATTAGCCCTTTTTTTCATGATTGAAAAT   2218
            ************************************************************

Dwarf       GCATAGCCTGAGAGAATGCAAAAAATAAAAGAATATATATGGTGTAATTGTCGAGTAAAA   2280
Tall        GCATAGCCTGAGAGAATGCAAAAAATAAAAGAATATATATGGTGTAATTGTCGAGTAAAA   2278
            ************************************************************

Dwarf       TAGAAAAGAGATAGTCGTATTATTTTTATAATTAGTGAACTTTTCGGGTA          2330
Tall        TAGAAAAGAGATAGTCGTATTATTTTTATAATTAGTGAACTTTTCGGGTA          2328
            **************************************************
```

Figure S2. The comparison of genomic DNA sequence of *CnHd3a* from tall and dwarf coconuts (CLUSTAL O (1.2.4) multiple sequence alignment). The grey colors indicate intron regions of conserved isoform. GT-AG splicing motifs are indicated by blue and red colors, respectively. *GT-AG* italic fronts indicate alternative donor and acceptor sites, respectively.

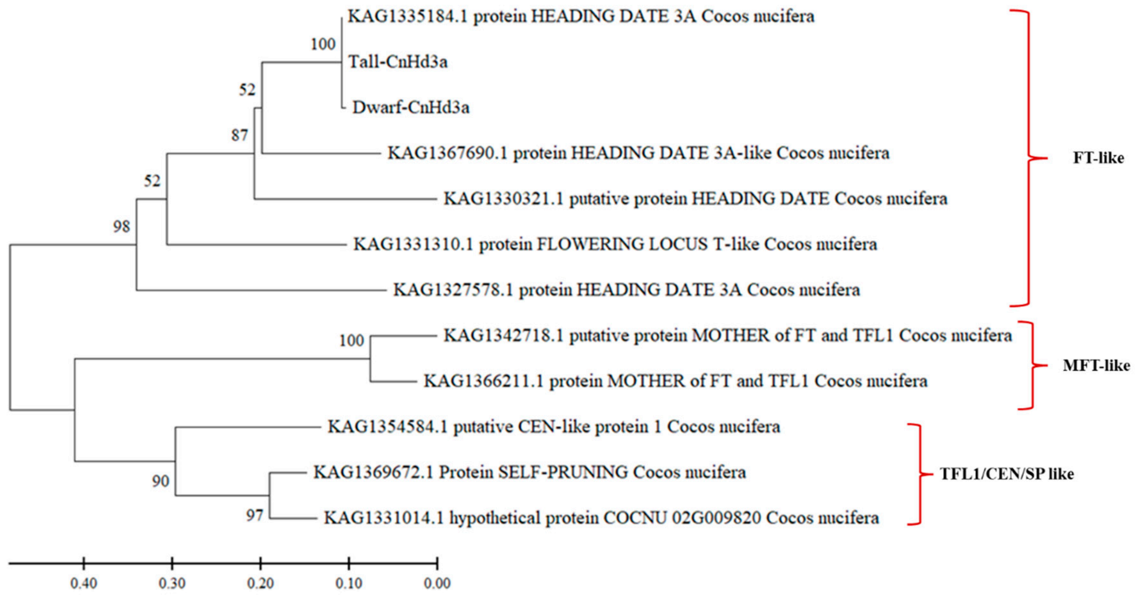

Figure S3. Phylogenetic tree of 10 predicted PEBP family members from coconut genome and CnHd3a of dwarf and tall coconuts. The tree was constructed using the ML method with 1,000 bootstrap replicates. Bootstrap values are indicated at the nodes. Evolutionary analyses were conducted in MEGA11 [50]

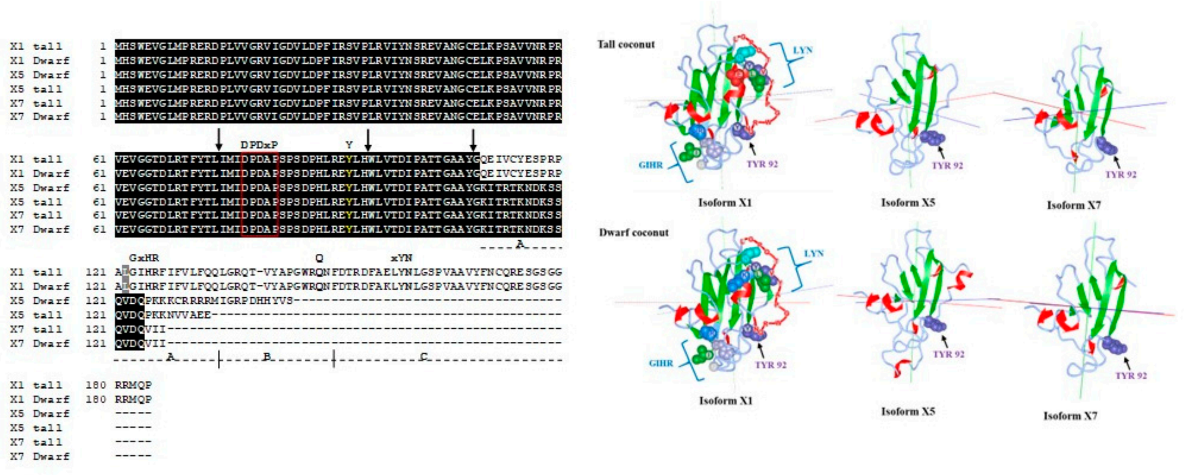

Figure S4. Multiple sequence alignment (A) and structural comparison of CnHd3a isoforms (X1, X5, and X7) from tall and dwarf coconut (B). Conserved motifs (DPDxP, GxHR, and xYN) and the critical residue Tyr-92 (Y) are annotated on the amino acid sequence, with Y highlighted in yellow. Black arrows mark the exon–exon junctions. Predicted 3D structures show the GIHR and LYN motifs labeled in blue, with secondary structures displayed as red α-helices, green β-sheets, and light blue loops. The critical residue Tyr-92 (Y) is also represented as an individual purple amino acid in the 3D structure. The external loop of segment B, highlighted in red with labeled amino acids, is present only in isoform X1 and absent in X5 and X7.
